# Determinants of Food Wasting Behavior: The Role of Organic Food Consumption and Environmental Literacy

**DOI:** 10.1002/fsn3.72210

**Published:** 2026-08-03

**Authors:** Sena Dilşad Akçakaya, Özge Mengi Çelik, Ayşe Kazanci, Sena Yildiz, Zeynep Aslı Beyazit, Burcu Sağlam

**Affiliations:** ^1^ Department of Nutrition and Dietetics, Gülhane Health Sciences Institute University of Health Sciences Ankara Turkey; ^2^ Department of Nutrition and Dietetics, Gülhane Health Sciences Faculty University of Health Sciences Ankara Turkey

**Keywords:** environmental literacy, food waste, organic food consumption

## Abstract

Mitigating the environmental impacts of the global food system while ensuring food security is a critical challenge, with food waste playing a central role. This study examines the relationships between organic food consumption, environmental literacy, and food wasting behaviors among adults. Through a web‐based cross‐sectional survey of 1025 adult participants, data were collected using demographic details, the Food Wasting Behaviors Questionnaire, the Organic Food Consumption Scale, and the Environmental Literacy Scale for Adults. Correlation and regression analyses revealed that higher organic food consumption was strongly associated with positive waste‐reduction practices, specifically buying as needed and planning shopping. Conversely, organic food consumption also served as a significant predictor for discarding unpalatable foods, indicating a higher propensity for waste generation among organic consumers, which may reflect a green licensing effect. In contrast, higher environmental literacy negatively predicted the tendency of discarding unpalatable foods while exhibiting strong positive relationships with circular waste behaviors, such as sharing food and feeding animals. Demographic analyses identified older age as a predictor of reduced food waste behaviors, whereas higher levels of education and income were associated with elevated waste tendencies. These relationships suggest that while cognitive environmental awareness is associated with active resource recovery, product‐centric sustainable choices can paradoxically relax behavioral discipline during the actual consumption stage. In conclusion, to mitigate food waste effectively, interventions must move beyond a sole focus on sustainable product selection; instead, policies should prioritize food management skills and enhance environmental literacy to prevent waste.

## Graphical abstract

Among 1025 adults, organic food consumption was associated with both positive waste‐reduction practices (buying as needed and planning shopping) and a greater likelihood of discarding unpalatable foods, suggesting a potential green licensing effect. Environmental literacy was associated with lower food discard and stronger circular waste behaviors (sharing food and feeding animals). Older age predicted lower food waste, whereas higher education and income predicted higher waste tendencies. Effective food‐waste prevention requires not only sustainable product choices but also stronger food management skills and environmental literacy.

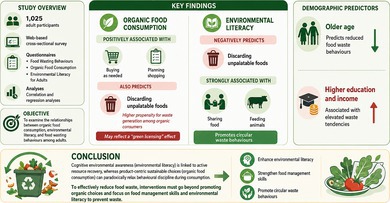

**Abbreviations**
BMI: Body Mass Index
ELSA: Environmental Literacy Scale for Adults
FWBQ: Food Wasting Behaviors Questionnaire
OFC: Organic Food Consumption Scale

## 1 Introduction

The global food system currently confronts a polycrisis era. This period is characterized by the intersection of the climate crisis, economic fluctuations, and intensifying social inequalities (Sachs et al. 2025; United Nations Department of Economic and Social Affairs 2023). Prevailing production and consumption models contribute to nearly 30% of global greenhouse gas emissions (Varzakas and Smaoui 2024). These models also act as a primary driver of biodiversity loss and natural resource depletion (von Braun et al. 2023). These ecological strains are further exacerbated by severe humanitarian crises. Currently, 733 million individuals suffer from chronic hunger, and 2.6 million people are unable to access healthy diets (Sachs et al. 2025; Hussain et al. 2025). This situation directly reflects the triple burden of malnutrition, which encompasses chronic hunger, micronutrient deficiencies, and obesity (Hussain et al. 2025). Such deep‐seated challenges underscore the critical necessity for a comprehensive transformation of food systems. Ultimately, future frameworks must integrate ethical, health, and governance perspectives rather than focusing on mere efficiency (Hussain et al. 2025; Shalini et al. 2025; Sousa et al. 2024).

Nearly one‐third of all food produced globally is wasted at the consumer or supply chain levels. This loss highlights a striking structural vulnerability within the current food system (Gage et al. 2024; United Nations Environment Programme 2024 2024). This inefficiency carries an annual cost exceeding 1 trillion dollars (United Nations Environment Programme 2024 2024). Furthermore, it signifies the squandering of vital land, water, and energy resources, thereby deepening ecological destruction (Varjani et al. 2024). Sustainable food system frameworks conceptualize the food system as a pursuit of equilibrium. Within this context, these frameworks emphasize the transformative potential of individual consumer behaviors (Price and Clark 2024). In particular, environmental literacy levels reflect the ecological knowledge and sensitivity of adults. Together with organic food consumption tendencies, these literacy levels serve as pivotal variables in regulating food wasting behaviors and fostering sustainable food systems (Attiq et al. 2021; Deliberador et al. 2024; Hamzaoğlu and Öztürk Göktuna 2022).

Current food systems are unsustainable. To facilitate change, individuals must develop a comprehensive environmental competence that transcends passive awareness. Within this framework, environmental literacy is recognized as a fundamental dynamic for transitioning to sustainable food systems. It represents a multi‐layered structure embodying the capacity to translate cognitive assets and affective values into tangible, responsible actions (Gray 2018; Hollweg et al. 2011). In this process, information is synthesized with attitudes to produce conscious preferences. Literacy functions not merely as a degree of awareness, but as a strategic roadmap for consumers to manage their ecological footprints (Kaya and Elster 2019; Lovren and Jablanovic 2023). Charles E Roth, a prominent theorist of the concept, characterizes environmental literacy beyond just being informed. He defines it as the ability to comprehend the interplay between environmental and human systems, enabling responsible conduct regarding environmental problems (Roth 1992). An individual possessing this perspective does not see food as a routine consumption item. Instead, they identify food as a component of an extensive energy and resource cycle spanning from production to the waste phase (Jurgilevich et al. 2016). This holistic outlook guides consumers toward production models with minimal environmental impacts. Furthermore, it acts as a vital driving force in minimizing post‐consumption losses through an internal sense of responsibility aimed at resource preservation (Aschemann‐Witzel et al. 2015).

Within sustainable consumer behavior research, these interactive pathways are analytically anchored in the Theory of Planned Behavior (Ajzen 1991) and the Value‐Belief‐Norm theory (Stern 2000). From this theoretical perspective, biosocial values and environmental literacy operate as foundational cognitive drivers that shape consumer attitudes and subjective norms during the purchasing phase, thereby predicting the specific intention toward organic food consumption (Gamage et al. 2023; Parashar et al. 2023). Organic food represents more than an agricultural product excluding synthetic chemicals and genetically modified organisms. Instead, it reflects a holistic production philosophy established upon protecting biodiversity, enhancing soil fertility, and preserving water resources (Joint FAO/WHO Codex Alimentarius Commission, Food and Agriculture Organization of the United Nations, and World Health Organization 2007; Paull 2010). Consumer orientation toward organic food is largely grounded in health motivations developed through perceptions of food safety and nutrition. Environmental anxiety can reinforce and shape these intentions, particularly through social norms and ecological values. From this perspective, organic products constitute an ethical consumption model. This model integrates the desire of the individual to protect both personal health and the health of the planet (Ahmed et al. 2021; Rana and Paul 2020; Teixeira et al. 2021; Xu et al. 2024). Furthermore, organic production requires intensive resource utilization and generates unique environmental value. This value perception can become a vital component of an integrated TPB strategy. Specifically, it directly targets consumer attitudes, subjective norms, and perceived behavioral control intended to minimize food waste (Etim et al. 2025). Based on these concepts, this value perception is not confined to the choice of the individual at the point of sale. Instead, it can also establish the basis for sensitivity concerning the management and prevention of waste within the domestic environment (Deliberador et al. 2024; Nadricka et al. 2024).

Prior studies have evaluated environmental literacy and organic consumption independently. However, their combined effects on specific sub‐dimensions of household food wasting behaviors remain largely unexamined. Furthermore, existing literature often overlooks the complexities of ‘green licensing effect’ (Khan and Dhar 2006; Mazar and Zhong 2010). This framework represents a critical behavioral paradox where initial moral or pro‐environmental choices, such as purchasing organic food, can unconsciously release a consumer's cognitive vigilance. This mechanism subsequently permits higher domestic waste generation, which manifests through behaviors like discarding unpalatable foods.

The primary objective of this research is to evaluate the direct relationships between environmental literacy, organic food consumption, and various food waste behaviors among adults. Specifically, the study addresses three key research questions (RQs):

- RQ1: What are the descriptive levels of environmental literacy and organic food consumption among adults?
- RQ2: How do environmental literacy and organic food consumption relate to distinct household food waste behaviors?
- RQ3: What is the predictive power of demographic factors, environmental literacy, and organic food consumption on household waste dimensions?

Global concerns regarding food security and environmental degradation are steadily growing. Consequently, this study fills a significant gap in the literature by linking psychological environmental factors with actual household food waste routines. The findings provide a clear framework for future longitudinal research. Additionally, they can guide the development of intervention studies and public health policies aimed at promoting sustainable nutrition behaviors.

## 2 Matherial and Methods

This research employed a cross‐sectional and descriptive study design. The study was conducted with a total of 1025 adult participants, comprising 619 females and 406 males aged between 19 and 65. All participants resided in the province of Ankara during the data collection period from September 15, 2025, to December 15, 2025. Data were gathered through a web‐based survey form developed via Google Forms. The recruitment process utilized the snowball sampling method. Social media platforms, including Instagram, X, Facebook, and WhatsApp, were employed to disseminate the questionnaire link. An a priori power analysis was performed using the G*Power software to estimate the required sample size. The analysis considered a statistical power of 80%, an alpha error coefficient of 0.05, and an expected correlation level of 0.30 between the instruments. Based on these parameters, the minimum target sample size was determined as 1020 volunteers. The inclusion criteria required participants to be between 19 and 65 years of age and to reside in Ankara. Additionally, individuals had to provide explicit digital consent by selecting the mandatory verification tab at the start of the survey. Ethical approval was obtained from the University of Health Sciences Gulhane Scientific Research Ethics Committee before the commencement of the research, with decision number 2025/469. All processes strictly adhered to the principles of the Declaration of Helsinki by the World Medical Association. The digital consent form explicitly outlined data confidentiality and the voluntary nature of participation. The survey collected demographic characteristics, including gender, age, education level, marital status, income level, and employment status. Anthropometric measurements of body weight and height were recorded concurrently via participant self‐reports. Additionally, the research investigated the relationships between organic food consumption, environmental literacy, and food wasting behaviors by employing the Organic Food Consumption Scale (OFC), the Environmental Literacy Scale for Adults (ELSA), and the Food Wasting Behaviors Questionnaire (FWBQ), which are validated in Turkish.

### 2.1 Anthropometric Measurements

Participants provided self‐reported data for body weight and height. The questionnaire included standardized instructions on how to record these measurements accurately. Body Mass Index (BMI) was calculated by dividing body weight (kg) by the square of height (m^2^). Weight status categories followed the World Health Organization criteria. A BMI below 18.50 kg/m^2^ was classified as underweight, 18.50–24.99 kg/m^2^ as normal weight, 25.00–29.99 kg/m^2^ as overweight, and 30.00 kg/m^2^ or higher as obese (WHO 2025).

### 2.2 Organic Food Consumption Scale (OFC)

The 18‐item OFC (Icli et al. 2019) was utilized to evaluate participants' orientations. Structurally, this measurement tool comprises six sub‐dimensions: Norms, Health Consciousness, Self‐Identity, Benefits, Positive Moral Attitude, and Information Seeking. It is based on a 5‐point Likert‐type rating ranging from 1 (Strongly Disagree) to 5 (Strongly Agree). Detailed information regarding the scale is provided in Appendix A (Tables A1, A2, A3, A4).

### 2.3 Environmental Literacy Scale for Adults (ELSA)

The ELSA (Atabek‐Yiğit et al. 2014) consists of 20 items distributed across three main dimensions: environmental consciousness, environmental anxiety, and environmental awareness. The instrument employs a 5‐point Likert‐type scoring system. Detailed information regarding the scale is provided in Appendix A.

### 2.4 Food Wasting Behaviors Questionnaire (FWBQ)

The FWBQ, was developed by Misiak et al. (Misiak et al. 2023) and validated in Turkish by Kendirli ve ark. (Kendirli et al. 2025). The questionnaire is scored on a 5‐point Likert‐type scale ranging from 1 (Never) to 5 (Always). Detailed information regarding the scale is provided in Appendix A.

### 2.5 Statistical Analysis

G*Power software (Version 3.1.9.6) was utilized to determine the sample size of the study. A medium effect size (|ρ| = 0.30) was assumed for the correlation between the FWBQ and ELSA. A power analysis was conducted using a two‐tailed hypothesis, a 5% alpha error probability, and 80% power (1‐β). Based on these parameters, the minimum required sample size was calculated as 1020. Ultimately, the study was completed with 1025 participants. Three specific RQs guided the analytical approach. The first question (RQ1) examined the descriptive levels of environmental literacy and organic food consumption among adults. Descriptive statistics answered RQ1 by calculating frequencies, percentages, means, and standard deviations. The second question (RQ2) evaluated how environmental literacy and organic food consumption relate to distinct household food waste behaviors. Pearson correlation coefficients addressed RQ2 by analyzing bivariate relationships. The third question (RQ3) investigated the predictive power of demographic factors, environmental literacy, and organic food consumption on household waste dimensions. Multiple linear regression models answered RQ3 to determine these predictive effects. All statistical analyses were performed using SPSS (Statistical Package for the Social Sciences) version 22.0 software. Descriptive statistics summarized the core data. Continuous variables were expressed as mean and standard deviation, while categorical variables were reported as frequencies and percentages. The normality of the data was evaluated via histograms, coefficient of variation, skewness, kurtosis values, and the Kolmogorov–Smirnov test. Pearson correlation coefficients were used to examine the bivariate relationships between variables. Additionally, Multiple Linear Regression analysis evaluated the predictive effects of demographic characteristics and scale scores on food wasting behaviors. Demographic predictors encompassed age, education level, marital status, and income status. Dependent variables included buying as needed, planning meals, planning shopping, discarding unpalatable foods, sharing food, and feeding animals. In all analyses, results were interpreted within a 95% confidence interval. The statistical significance level was accepted as *p* < 0.05.

## 3 Results

This study was conducted among 1025 adults (619 females, 406 males). The general characteristics of the participants are shown in Table 1. The majority of the participants were female (60.4%). The mean age was 29.1 ± 10.5 years, and the mean BMI was 24.5 ± 3.8 kg/m^2^. Regarding educational status, 66.9% were university graduates, and regarding marital status, 70.0% were single. In terms of socioeconomic status, 30.2% of the participants reported income less than their expenses, while 24.7% had income exceeding their expenses. More than half of the participants (55.0%) were unemployed at the time of the study. According to the Body Mass Index (BMI) classification, 3.4% of the participants were underweight, 55.8% were normal weight, 32.7% were overweight, and 8.1% were obese. The descriptive statistics, including the mean scores obtained from the sub‐dimensions of the FWBQ, the OFC total score, and the ELSA total score along with its sub‐dimensions, are comprehensively detailed in Table 1.

**TABLE 1:** General characteristics of individuals.

Variables	$\bar{X}$±SD
Age (year)	29.1 ± 10.5
BMI (kg/m^2^)	24.5 ± 3.8
Food Wasting Behaviors Questionnaire	
Discarding unpalatable foods	25.4 ± 9.6
Buying as needed	32.3 ± 7.8
Sharing food	28.5 ± 9.7
Planning meals	10.3 ± 5.5
Planning shopping	14.3 ± 5.1
Feeding animals	13.2 ± 6.2
Organic Food Consumption Scale	
Total score	63.4 ± 13.7
Norms	12.8 ± 3.8
Health consciousness	11.1 ± 2.6
Self‐Identity	10.4 ± 2.6
Benefits of consuming organic food	14.8 ± 3.6
Positive moral attitudes	7.4 ± 1.8
Information‐seeking	6.7 ± 2.1
Environmental Literacy Scale for Adults (ELSA)	
Total score	78.2 ± 12.7
Environmental consciousness level	37.5 ± 5.7
Environmental anxiety level	24.3 ± 4.4
Environmental awareness level	16.3 ± 5.1
	*N* (%)
Gender	
Female	619 (60.4)
Male	406 (39.6)
Education Level	
Primary school	15 (1.5)
Middle school	241 (23.5)
High school	30 (2.9)
University	686 (66.9)
Master's degree/Doctorate	53 (5.2)
Working status	
Yes	461 (45.0)
No	564 (55.0)
Income status	
Income less than expenses	310 (30.2)
Income equal to expenses	462 (45.1)
Income more than expenses	253 (24.7)
Marital status	
Single	717 (70.0)
Married	308 (30.0)
BMI classification	
Underweight (< 18.50 kg/m^2^)	35 (3.4)
Normal (18.50–24.99 kg/m^2^)	572 (55.8)
Overweight (25.00–29.99 kg/m^2^)	335 (32.7)
Obese (≥ 30.0 kg/m^2^)	83 (8.1)
Number of main meals	2.4 ± 0.5
Number of snacks	1.3 ± 0.8

**FIGURE 1:**
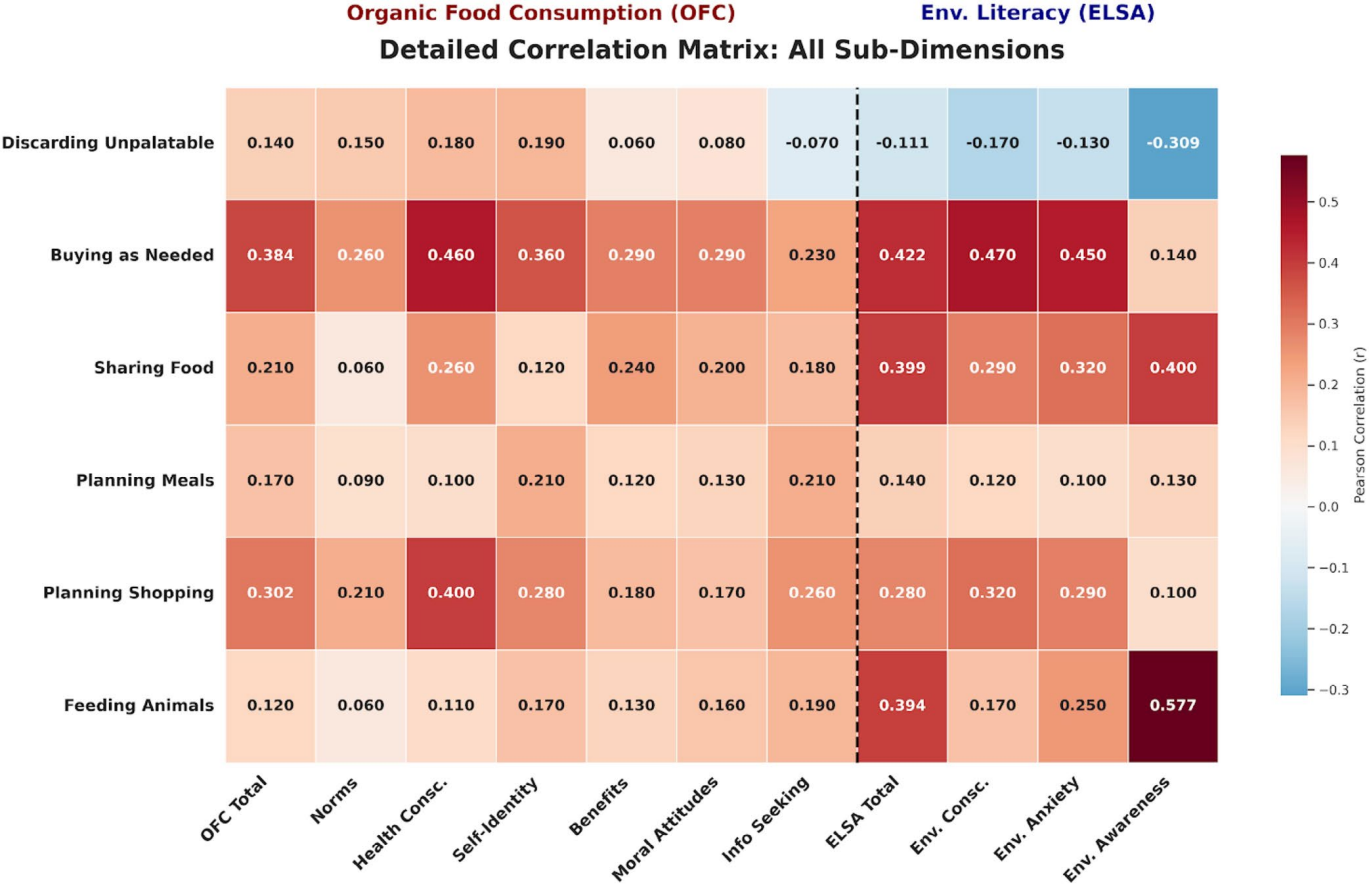
Detailed correlation matrix across total scores and sub‐dimensions of organic food consumption, environmental literacy, and food wasting behaviors.

The relationships between the OFC, the ELSA, and the FWBQ sub‐dimensions are presented in Table 2. The OFC total score correlated positively and significantly with all sub‐dimensions of the FWBQ (*p* < 0.001). Specifically, positive correlations occurred between the OFC total score and the scores for buying as needed, planning shopping, and sharing food. Regarding the ELSA, the total score exhibited a positive and significant correlation with most food wasting behavior sub‐dimensions (*p* < 0.001). These associations involved the dimensions of buying as needed, sharing food, and feeding animals. Conversely, the ELSA total score correlated negatively and significantly with discarding unpalatable foods (*p* < 0.001). Among the sub‐dimensions, the environmental awareness level showed a positive relationship with feeding animals and a significant negative relationship with discarding unpalatable foods (*p* < 0.001). Figure 1 presents the correlation matrix across the sub‐dimensions of organic food consumption, environmental literacy, and food wasting behaviors.

**TABLE 2:** The relationship between food wasting behaviors questionnaire, organic food consumption scale, and environmental literacy scale for adults.

	Organic Food Consumption Scale							Environmental Literacy Scale for Adults (ELSA)			
	Total score	Norms	Health consciousness	Self‐Identity	Benefits of consuming organic food	Positive moral attitudes	Information‐seeking	Total score	Environmental consciousness level	Environmental anxiety level	Environmental awareness level
Food Wasting Behaviors Questionnaire											
Discarding	*r* = 0.135	*r* = 0.151	*r* = 0.180	*r* = 0.192	*r* = 0.061	*r* = 0.079	*r* = −0.071	*r* = −0.111	*r* = −0.165	*r* = −0.126	*r* = −0.309
Unpalatable foods	*p* < 0.001*	*p* < 0.001*	*p* < 0.001*	*p* < 0.001*	*p* = 0.049*	*p* = 0.011*	*p* = 0.020*	*p* < 0.001*	*p* < 0.001*	*p* < 0.001*	*p* < 0.001*
Buying as needed	*r* = 0.384 *p* < 0.001*	*r* = 0.258 *p* < 0.001*	*r* = 0.459 *p* < 0.001*	*r* = 0.362 *p* < 0.001*	*r* = 0.292 *p* < 0.001*	*r* = 0.294 *p* < 0.001*	*r* = 0.232 *p* < 0.001*	*r* = 0.422 *p* < 0.001*	*r* = 0.466 *p* < 0.001*	*r* = 0.454 *p* < 0.001*	*r* = 0.141 *p* < 0.001*
Sharing food	*r* = 0.210 *p* < 0.001*	*r* = 0.061 *p* = 0.049	*r* = 0.256 *p* < 0.001*	*r* = 0.124 *p* < 0.001*	*r* = 0.241 *p* < 0.001*	*r* = 0.203 *p* < 0.001*	*r* = 0.183 *p* < 0.001*	*r* = 0.399 *p* < 0.001*	*r* = 0.286 *p* < 0.001*	*r* = 0.321 *p* < 0.001*	*r* = 0.399 *p* < 0.001*
Planning meals	*r* = 0.166 *p* < 0.001*	*r* = 0.087 *p* = 0.005*	*r* = 0.101 *p* = 0.001*	*r* = 0.212 *p* < 0.001*	*r* = 0.117 *p* < 0.001*	*r* = 0.130 *p* < 0.001*	*r* = 0.210 *p* < 0.001*	*r* = 0.140 *p* < 0.001*	*r* = 0.120 *p* < 0.001*	*r* = 0.099 *p* = 0.002*	*r* = 0.129 *p* < 0.001*
Planning shopping	*r* = 0.302 *p* < 0.001*	*r* = 0.213 *p* < 0.001*	*r* = 0.400 *p* < 0.001*	*r* = 0.279 *p* < 0.001*	*r* = 0.179 *p* < 0.001*	*r* = 0.170 *p* < 0.001*	*r* = 0.263 *p* < 0.001*	*r* = 0.285 *p* < 0.001*	*r* = 0.324 *p* < 0.001*	*r* = 0.292 *p* < 0.001*	*r* = 0.096 *p* = 0.002*
Feeding animals	*r* = 0.125 *p* < 0.001*	*r* = 0.061 *p* = 0.049*	*r* = 0.106 *p* = 0.001*	*r* = 0.170 *p* < 0.001*	*r* = 0.177 *p* < 0.001*	*r* = 0.159 *p* < 0.001*	*r* = 0.188 *p* < 0.001*	*r* = 0.394 *p* < 0.001*	*r* = 0.165 *p* < 0.001*	*r* = 0.254 *p* < 0.001*	*r* = 0.577 *p* < 0.001*

* *p* < 0.05.

**FIGURE 2:**
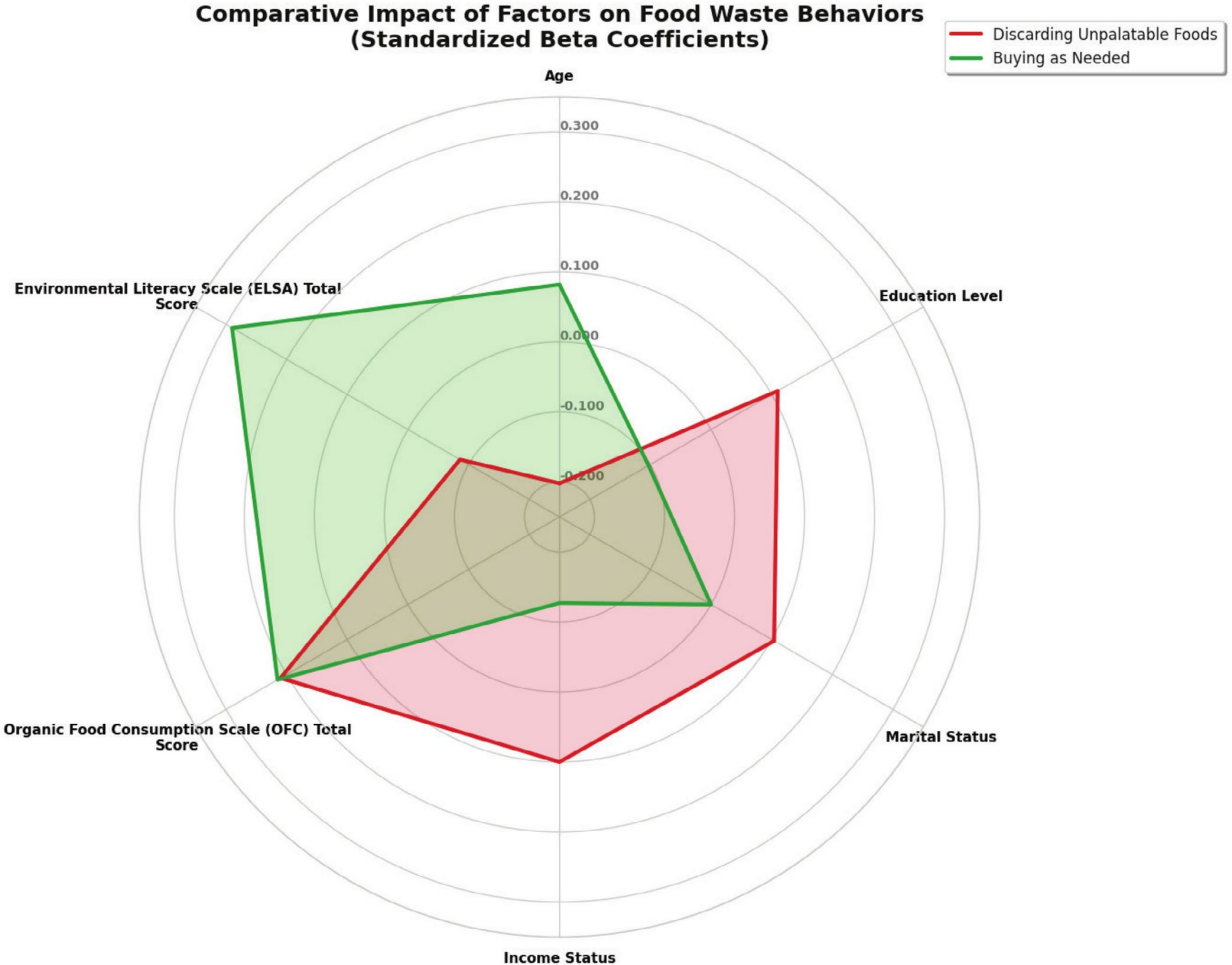
Comparative impact analysis of factors predicting discarding unpalatable foods and buying as needed behaviors based on standardized beta coefficients.

**FIGURE 3:**
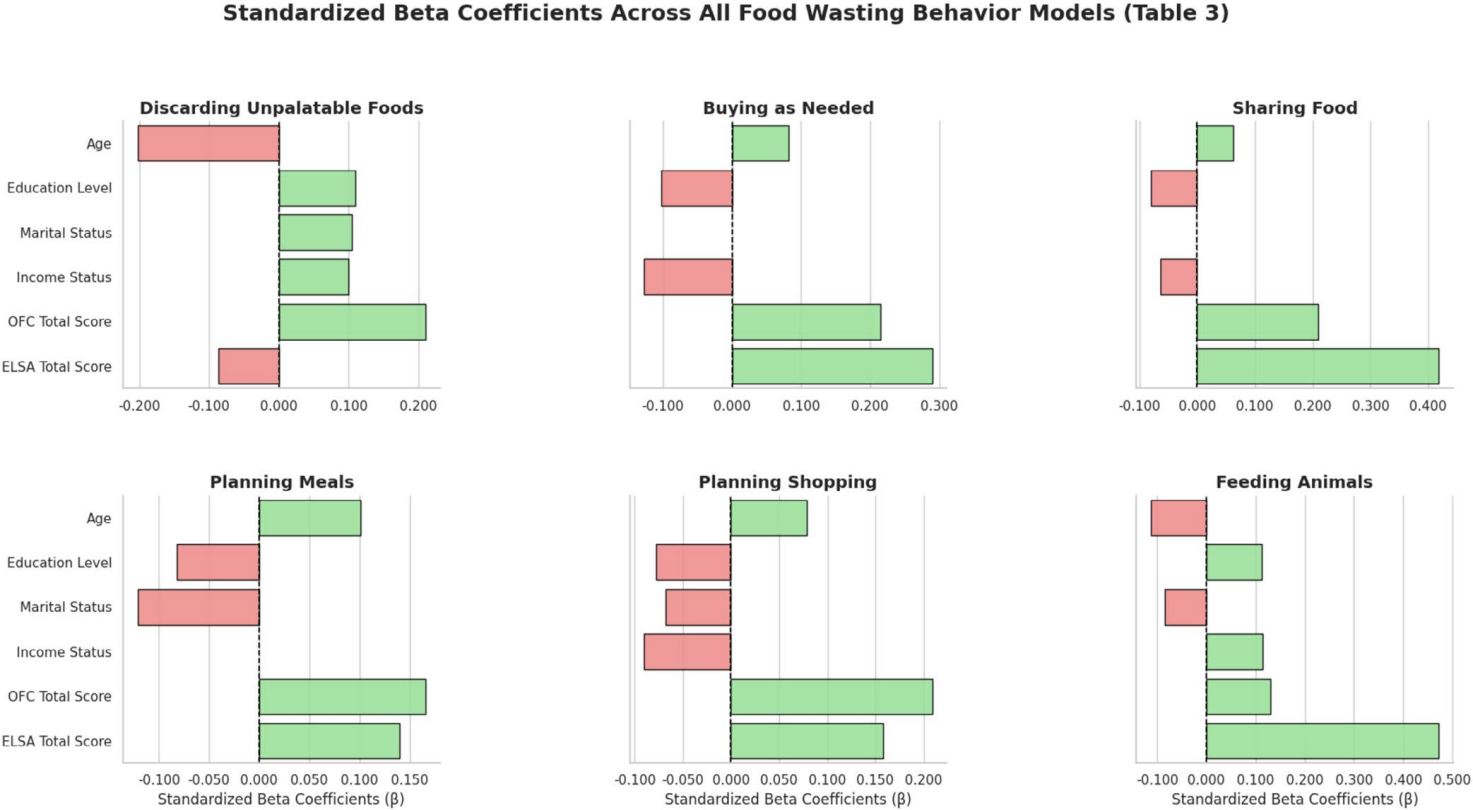
Standardized beta coefficients from multiple linear regression models predicting six sub‐dimensions of food wasting behaviors (*N* = 1025). Green bars indicate positive predictors, while red bars indicate negative predictors. Independent variables include age, education level, marital status, income status, and total scores from the OFC and ELSA. All values are reported to three decimal places (*p* < 0.05 for all highlighted variables).

The results of the linear regression analyses predicting the various sub‐dimensions of the FWBQ are presented in Table 3. The regression model predicting the discarding unpalatable foods score was statistically significant (*p* < 0.001). Within this model, age, education level, marital status, income status, OFC total score, and ELSA total score were significantly associated with the dependent variable (*p* < 0.05). Similarly, the independent regression models predicting buying as needed, sharing food, planning meals, planning shopping, and feeding animals were all statistically significant (*p* < 0.001). Figure 2 visualizes the relative impact of demographic characteristics and scale scores on discarding unpalatable foods and buying as needed, based on the standardized beta coefficients presented in Table 3. Additionally, Figure 3 presents a multi‐panel horizontal bar chart visualizing the standardized beta coefficients across all six food management dimensions. This chart illustrates the respective positive (green bars) and negative (red bars) effects of the independent variables for each model.

**TABLE 3:** Linear regression analyzes for organic food consumption and environmental literacy prediction.

Model	Unstandartized B	Standardized Beta	*t*	*p* value
Discarding unpalatable foods				
Age (year)	−0.184	−0.202	−6.462	**< 0.001** *
Education level	1.572	0.110	3.382	**0.001** *
Marital status	2.180	0.104	2.491	**0.013** *
Income status	1.294	0.100	2.864	**0.004** *
OFC total score	0.147	0.210	5.839	**< 0.001** *
ELSA Total Score	−0.065	−0.086	−2.369	**0.018** *
	**R** ^**2**^ **= 0.312; *p* < 0.001** *			
Buying as needed				
Age (year)	0.061	0.082	2.088	**0.037** *
Education level	−1.190	−0.103	−3.466	**0.001** *
Income status	−1.340	−0.127	−4.313	**< 0.001** *
OFC Total Score	0.123	0.215	6.493	**< 0.001** *
ELSA Total Score	0.178	0.290	8.642	**< 0.001** *
	**R** ^**2**^ **= 0.264; *p* < 0.001** *			
Sharing food				
Age (year)	0.059	0.063	2.025	**0.043** *
Education level	−1.159	−0.080	−2.601	**0.009** *
Income status	−0.834	−0.063	−2.024	**0.043** *
OFC Total Score	0.150	0.210	6.881	**< 0.001** *
ELSA Total Score	0.319	0.418	11.959	**< 0.001** *
	**R** ^**2**^ **= 0.208; *p* < 0.001** *			
Planning meals				
Age (year)	0.053	0.101	3.239	**0.001** *
Education level	−0.670	−0.082	−2.494	**0.013** *
Marital status	−1.452	−0.121	−2.837	**0.005** *
OFC Total Score	0.067	0.166	5.395	**< 0.001** *
ELSA Total Score	0.060	0.140	4.517	**< 0.001** *
	**R** ^**2**^ **= 0.376; *p* < 0.001** *			
Planning shopping				
Age (year)	0.038	0.079	2.525	**0.012** *
Education level	−0.586	−0.078	−2.470	**0.014** *
Marital status	−0.754	−0.068	−2.192	**0.029** *
Income status	−0.618	−0.090	−2.880	**0.004** *
OFC Total Score	0.077	0.209	5.896	**< 0.001** *
ELSA Total Score	0.063	0.158	4.399	**< 0.001** *
	**R** ^**2**^ **= 0.216; *p* < 0.001** *			
Feeding animals				
Age (year)	−0.067	−0.113	−2.823	**0.005** *
Education level	1.049	0.113	3.745	**< 0.001** *
Marital status	−1.156	−0.085	−2.167	**0.030** *
Income status	0.971	0.115	3.834	**< 0.001**
OFC Total Score	0.059	0.130	3.810	**< 0.001** *
ELSA Total Score	0.230	0.472	13.706	**< 0.001** *
	**R** ^**2**^ **= 0.278; *p* < 0.001** *			

*Note:* Variable values: ducation level (Primary school graduate = 1, Middle school graduate = 2, High school graduate = 3, University graduate = 4, Master's and Doctorate graduates = 5), Income Status (Income less than expenses = 1, Income equal to expenses = 2, Income more than expenses = 3), Marital status (Married = 0, Single = 1).

* *p* < 0.05.

Organic food consumption and environmental literacy levels positively predicted the scores for buying as needed, sharing food, planning meals, planning shopping, and feeding animals (*p* < 0.05) (Table 3). Within these models, the ELSA score exhibited the highest standardized beta values for sharing food and feeding animals. For the discarding unpalatable foods model, the OFC total score acted as a positive predictor, whereas the ELSA total score served as a negative predictor. Regarding demographic variables, age positively predicted planning and buying scores. These directional effects across all six regression models are visually summarized in Figure 3.

## 4 Discussion

The primary objective of this study was to evaluate the relationships between environmental literacy, organic food consumption, and distinct household food wasting behaviors among adults. The structured outcomes establish a clear hierarchy among these behavioral determinants. Environmental literacy serves as the primary negative predictor of waste across domestic routines. Demographic attributes modulate these sub‐dimensional behaviors as secondary factors. Finally, a distinct behavioral paradox emerges regarding organic food consumption, which simultaneously predicts disciplined purchasing habits and an increased tendency to discard unpalatable foods.

Regarding the paradoxical findings of the model, the behavioral configurations of sustainable consumers reveal a complex friction between proactive procurement and actual domestic waste outcomes, supporting the theoretical framework of ElHaffar et al. (ElHaffar et al. 2020). The outcomes indicate that organic food consumption aligns positively with highly disciplined purchasing phases, specifically focusing on buying as needed and planning shopping routines. This alignment supports the premise that organic consumers maintain elevated environmental motivations that translate into structured management prior to consumption (Ahmed et al. 2021; Aprile and Fiorillo 2023; Janssens et al. 2019; Mengi Çelik, Akçakaya, and Ekici 2025; Pilone et al. 2023). Conversely, the analysis exposes a critical divergence: higher orientations toward organic food consumption positively predict the tendency to discard unpalatable foods. This phenomenon, explained in the literature through the concept of green licensing, points to the risk that an individual's eco‐friendly attitude in one domain (e.g., purchasing organic products) may psychologically rationalize and justify irresponsible behavior in another (Mazar and Zhong 2010). This tendency is attributed to various technical and psychological dynamics within the literature. From a technical perspective, the absence of synthetic preservatives and pesticide residues in organic products accelerates biochemical degradation processes compared to conventional alternatives, which is thought to trigger higher waste rates (Chauhan and Rao 2024; Karanth et al. 2023). While some research argues that specific organic products may offer longer storage life under ideal conditions (Alessandroni et al. 2024; Csambalik et al. 2023), household data document higher waste rates due to rapid perishability (Eriksson et al. 2014). Beyond cosmetic aesthetics, because organic farming avoids chemical enhancements, fruits and vegetables frequently deviate from visually perfect standards (Puteri et al. 2022). Health‐conscious consumers may perceive even the slightest wilting or color change in these products as a direct food safety risk, leading to a tendency to quickly discard the item or avoid purchasing it altogether (Dusoruth and Peterson 2020; Hingston and Noseworthy 2020). This aversion is further compounded by misleading perceptions that organic food waste is less harmful to the environment, which weakens the motivation to rescue imperfect products and reduces waste sensitivity (Nadricka et al. 2024). The expectation of a flawless sensory experience among consumers who pay a price premium for organic food creates a contradiction when products appear unpalatable or bruised. This mismatch with the price‐quality balance results in waste, both through low purchase intent and by triggering discard behavior within the home (Melovic et al. 2020; Oktaviani et al. 2024; Szymkowiak et al. 2022). Aligned with these observations, the study confirmed that organic food consumers act consciously during the purchasing phase, demonstrating strong correlations with buying as needed and planning shopping. However, the statistical analyses also revealed that high levels of organic food consumption act as a significant positive predictor of discarding unpalatable foods. These results confirm the dual role of organic food consumption: while an orientation toward organic food encourages regular shopping habits and planning, it may prove insufficient in preventing waste during the consumption stage when products fail to meet taste or freshness expectations.

Regarding the primary findings of the model, environmental literacy serves as a prominent inverse predictor against domestic food waste. This direction aligns with broader sustainability literature documenting that eco‐literacy operates as a core protective asset in household resource management (Attiq et al. 2021; Ariyani and Ririh 2020; Kritikou et al. 2021). Literature indicates that individuals with high environmental literacy are consumers who comprehend ecosystem functioning, possess a high awareness of their ecological footprint, and integrate this awareness with ethical responsibility (Cooreman‐Algoed et al. 2021; Mengi Çelik, Ekici, et al. 2025; Yildirim et al. 2025). For these individuals, wasting food is perceived not merely as an economic loss but as an ecological system failure (Hollweg et al. 2011; Roth 1992). Investigations emphasize that increasing levels of environmental consciousness are closely associated with alternative recovery pathways instead of discarding food in the trash (Dusoruth and Peterson 2020; Kritikou et al. 2021). Knowing the environmental benefits and practical methods of waste reduction is documented to support preventive behaviors, such as utilizing food waste as animal feed (Parsa et al. 2023; Srijuntrapun et al. 2025). Romani et al. revealed that environmental awareness and the intention to prevent waste alone are insufficient; food‐related routines such as pre‐shopping planning, meal preparation, and leftover management are decisive in translating intention into behavior (Romani et al. 2018). In this context, the lack of planning meals represents a critical vulnerability associated with elevated waste, whereas educational interventions aimed at improving this skill have been shown to significantly reduce domestic food waste. As awareness regarding the wide‐ranging consequences of food waste increases, the likelihood of adopting preventive behaviors such as planning shopping and minimizing waste is seen to rise (Principato et al. 2015). Individuals who maintain consistent routines, such as pre‐shopping stock control and list‐keeping (referred to as shopping planners) are characterized as the group with the highest food management skills and awareness. The active participation of these individuals in recycling activities enhances their awareness of the causes of food waste, correlating with lower domestic waste levels (Dusoruth and Peterson 2020). The outcomes align with this empirical framework, demonstrating that environmental literacy operates as a negative predictor for discarding unpalatable foods while positively predicting proactive dimensions like buying as needed and planning meals. Notably, eco‐awareness exhibits its strongest predictive weight on alternative recovery loops (specifically sharing food and feeding animals). These results suggest that environmental consciousness directs the individual not only toward the passive act of not discarding food but toward active solution strategies that keep food within the ecosystem.

In terms of the secondary findings of the model, age operates as the primary demographic predictor of domestic food wasting behaviors. The findings indicate that the age variable serves as a negative predictor against the tendency toward discarding unpalatable foods. This aligns with the broad literature consensus suggesting that the propensity for food waste systematically decreases as age increases (Dusoruth and Peterson 2020; Kritikou et al. 2021; Principato et al. 2015; Gimenez et al. 2023; Grasso et al. 2019; Melbye et al. 2017; Stoica et al. 2025). Indeed, Kritikou et al. (Kritikou et al. 2021), state that older individuals experience a higher sense of moral obligation regarding food management and categorize wasting food as an intolerable behavior. On a similar note, Przezbórska‐Skobiej and Wiza (Przezbórska‐Skobiej and Wiza 2021), emphasize that while youth exhibit a more impulsive profile in food management, the older age group acts with significantly greater discipline in shopping list preparation and food storage processes. This disparity is often pronounced in younger cohorts, specifically Gen Y and Gen Z, who frequently possess weaker food preparation skills and less experience in kitchen management, where the time‐poverty constraints of these specific demographics correlate with unplanned consumption and consequently higher waste rates (Dusoruth and Peterson 2020; Principato et al. 2015). The outcomes correspond with this theoretical framework, showing that advancing age negatively predicts waste tendencies while positively predicting proactive behavior patterns across buying as needed and planning routines. This pattern suggests that as age increases, accumulated kitchen experience and shifting moral priorities might more effectively translate sustainable intentions into disciplined household management, a capacity that younger cohorts may still be developing.

In addition to age, the analysis demonstrates a positive relationship between education level and the tendency toward discarding unpalatable foods. Within the academic literature, there is no consensus regarding the direction of the association between education level and waste. While Srijuntrapun et al. (Srijuntrapun et al. 2025); argue that increasing education levels reduce waste by fostering more competent waste management practices, several studies indicate that higher waste is observed among highly educated and high‐income groups due to greater purchasing power (Grasso et al. 2019; Stoica et al. 2025; Li et al. 2023; Lima et al. 2024) Stoica et al. (Stoica et al. 2025) further supported that highly educated individuals typically possess higher income levels and attributed the elevated waste generation to the time poverty phenomenon resulting from intensive professional lives. Such constraints may align educated individuals with practical yet waste‐intensive buy‐and‐discard behaviors rather than engaging in kitchen planning and food evaluation processes. The results extend this viewpoint, indicating that higher formal education does not automatically ensure waste mitigation unless accompanied by practical time‐allocation strategies for household routines.

A similar dynamic was identified regarding the income variable; as income levels rise, the likelihood of discarding unpalatable food increases. Przezbórska‐Skobiej and Wiza (Przezbórska‐Skobiej and Wiza 2021), ground this finding in the low economic value theory: while low‐income households utilize food rationally and cautiously due to budget constraints, the diminishing share of food expenditures within the total budget of high‐income households alleviates financial concern, which is psychologically associated with higher waste. Numerous studies further indicate that waste increases alongside rising income because the economic significance of food diminishes (Srijuntrapun et al. 2025; Li et al. 2023; Lima et al. 2024). This outcome suggests that higher income levels may relax the domestic budget constraint in the examined sample, potentially lowering the perceived cognitive and financial cost of discarding unpalatable items.

As part of the secondary demographic outcomes of the model, marital status emerges as a further factor, where being married positively relates to higher domestic waste tendencies compared to single individuals. Although some studies in the literature suggest that per capita waste amounts are generally higher in smaller or single‐person households due to economies of scale (Gimenez et al. 2023; Herzberg et al. 2020; Koivupuro et al. 2012), substantial evidence indicates that total waste volume can be higher in large families and households with children, where over‐purchasing and portioning issues are prevalent (Stoica et al. 2025; Ilakovac et al. 2020; Tonini et al. 2023). Literature emphasizes that the diversity in consumer preferences and planning challenges, such as unpredictable meal cancellations, frequently correlate with higher waste levels in multi‐person domestic units (Stoica et al. 2025). These collective demographic indicators suggest that food waste mitigation strategies aimed at higher socio‐economic and married cohorts might be more effectively structured around ethical responsibility and time‐management frameworks rather than economic savings incentives.

In synthesis, the outcomes of this study demonstrate the combined interactions of psychological, behavioral, and demographic vectors within household food management. Environmental literacy operates as a foundational cognitive factor that predicts proactive planning and circular waste mitigation routines, aligning with the cognitive‐to‐behavioral trajectories of the Value‐Belief‐Norm theory (Stern 2000). Conversely, organic food consumption exhibits a dual orientation. It predicts disciplined purchasing habits but also associates with higher discard tendencies during the domestic consumption stage due to heightened aesthetic and freshness sensitivities, validating the psychological boundaries of the green licensing framework (Khan and Dhar 2006; Mazar and Zhong 2010). Demographic attributes modulate these trajectories as conditional factors. Advancing age relates to higher behavioral discipline. In contrast, higher socioeconomic status and time‐poverty constraints align with increased waste tendencies. These interactive pathways suggest that comprehensive waste reduction requires policy interventions that transition from simple product choices to structured kitchen management skills across diverse demographic cohorts.

This research contributes to consumer sustainability literature by evaluating the multidimensional interactions between organic food consumption, environmental literacy, and food wasting behaviors. A key strength involves elucidating the role of environmental literacy across domestic routines, particularly regarding alternative recovery pathways like sharing food and feeding animals. The sample size of *N* = 1025 participants further supports the statistical reliability of the regression models. Several limitations warrant consideration when evaluating these findings. Primarily, the cross‐sectional design precludes drawing definitive causal inferences among the studied variables. Consequently, future longitudinal research is essential to clarify the exact directionality of these relationships. Regarding sampling methodology, the utilization of a web‐based survey and snowball sampling introduces selection bias. The resulting sample is skewed toward younger, highly educated, and digitally literate individuals. Consequently, the domestic behaviors of this specific cohort interact closely with severe time‐poverty constraints. This reality varies from a broader national average where traditional, slower food management routines may yield lower structural waste. This baseline profile can also result in an over‐representation of participants already familiar with sustainability concepts. Future investigations should employ diversified data collection methodologies to enhance population generalizability. Regarding measurement parameters, the reliance on self‐reported questionnaires introduces potential skew. For instance, self‐reported height and weight for BMI calculations remain susceptible to reporting bias. Similarly, measuring ecologically sensitive variables via self‐report makes the data vulnerable to social desirability bias. Participants may unconsciously over‐report environmental literacy levels to align with ecological norms while under‐reporting actual household discard behaviors. This potential variance implies that the observed gap between environmental awareness and consumption‐stage waste might be even wider in unmonitored settings. Finally, while the regression models are statistically significant, a portion of the variance in food wasting behavior remains unexplained. This highlights the need for future research to incorporate additional determinants, such as situational household logistics and specific psychological traits to explain the remaining variance in the regression models. Future studies would also benefit from incorporating exhaustive economic analyses regarding purchasing power and price premiums to evaluate financial constraints more deeply.

## 5 Conclusion

This study examines the relationships between organic food consumption, environmental literacy, and domestic food wasting behaviors among adults. The outcomes indicate that organic food consumption shows a dual orientation. It is associated with disciplined behavior during the purchasing phase but relates positively to discard tendencies at the consumption stage. This pattern highlights a green licensing mechanism where pro‐environmental purchase choices inadvertently relax domestic waste vigilance. Concurrently, environmental literacy operates as an inverse predictor against household food waste. Higher eco‐literacy scores are associated with active circular waste strategies, including sharing food and feeding animals. Regarding demographic parameters, younger, highly educated, and high‐income cohorts display higher vulnerabilities to specific waste routines. This suggests that waste tendencies are closely associated with managerial and temporal constraints rather than purely financial vectors. Consequently, public health and environmental policies should move beyond generic awareness campaigns. Interventions should prioritize practical kitchen management and time‐allocation skills tailored for time‐poor consumer segments. Policy frameworks within sustainable programs could integrate targeted storage guidelines. These practical tools can support organic consumers in managing rapid product perishability without triggering discard behaviors.

This study has certain methodological limitations that require cautious interpretation. The cross‐sectional design cannot establish permanent causal relationships among the variables. The online snowball sampling approach also limits generalizability, as the sample tracks with younger and highly educated adults. Furthermore, relying on self‐reported data might introduce social desirability bias. Future research should utilize longitudinal designs to monitor how household waste behaviors shift over time. Researchers should also incorporate direct waste observations or kitchen diaries to reduce reporting bias. Finally, introducing economic factors like purchasing power and price premiums will provide a deeper view of consumer choices.

## Author Contributions

**Özge Mengi Çelik:** conceptualization, investigation, writing – original draft, writing – review and editing, formal analysis, supervision. **Zeynep Aslı Beyazit:** conceptualization, investigation, writing – original draft, writing – review and editing. **Ayşe Kazanci:** investigation, conceptualization, writing – original draft, writing – review and editing. **Sena Dilşad Akçakaya:** investigation, conceptualization, writing – original draft, writing – review and editing. **Burcu Sağlam:** conceptualization, investigation, writing – original draft, writing – review and editing. **Sena Yildiz:** conceptualization, investigation, writing – original draft, writing – review and editing.

## Funding

The authors have nothing to report.

## Ethics Statement

All procedures performed in studies involving human participants were in accordance with the ethical standards of the institutional research committee and with the 1964 Helsinki declaration and its later amendments or comparable ethical standards. The study was conducted with the approval of the University of Health Sciences Gulhane Scientific Research Ethics Committee, numbered 2025/469.

## Consent

The authors have nothing to report.

## Conflicts of Interest

The authors declare no conflicts of interest.

## Data Availability

The data that support the findings of this study are available on request from the corresponding author. The data are not publicly available due to privacy or ethical restrictions.

## Appendix A Appendix Evaluation Instruments and Item Inventories

**TABLE A1:** Psychometric properties and operational details of the scales.

Scale/Construct	Sub‐dimensions	Number of items	Response format	Cronbach's Alpha (Current Study/Original)
OFC (Icli et al. 2019)	Norms, Health Consciousness, Self‐Identity, Benefits, Positive Moral Attitude, Information Seeking	18	5‐point Likert (1–5)	0.771 (Original Study)
ELSA (Atabek‐Yiğit et al. 2014)	Environmental Consciousness Environmental Anxiety Environmental Awareness	20	5‐point Likert (1–5)	Overall: 0.881 Dim 1: 0.807 Dim 2: 0.765 Dim 3: 0.715
FWBQ (Misiak et al. 2023; Kendirli et al. 2025)	Buying as needed Planning shopping Discarding unpalatable foods Sharing food Feeding animals	21	5‐point Likert (1–5)	0.758 0.884 0.908 0.824 0.851

### Full Inventory of Attitude Statements and Scale Items

**TABLE A2:** Items and sub‐dimensions of the Organic Food Consumption Scale (OFC) (Icli et al. 2019).

**Norms**
Item 1: Most people I know buy organic food.
Item 2: Friends buy organic food.
Item 3: People you know, buy organic food.
Item 4: People who are important to me would think I should buy organic food.
**Health Consciousness**
Item 5: I'm usually aware of my health.
Item 6: I'm alert to changes in my health.
Item 7: I reflect about my health a lot.
**Self‐Identity**
Item 8: I think of myself as someone who is concerned about the environmentalist nature of what I eat.
Item 9: I think of myself as someone who is concerned about health consequences of what I eat.
Item 10: I think of myself as someone who is concerned about the safety of what I eat.
**Benefits of Consuming Organic Food**
Item 11: For me, consuming organic food would be beneficial.
Item 12: For me, consuming organic food would be tasteful.
Item 13: For me, consuming organic food would be good.
Item 14: For me, consuming organic food would be valuable.
**Positive Moral Attitudes**
Item 15: Buying organic food instead of conventional ones would feel like making a personal contribution to something better.
Item 16: Buying organic food instead of conventional ones would make me feel that I am looking after my family better.
**Information‐seeking**
Item 17: I have informed myself about how to cook organic food at home.
Item 18: I have informed myself about how to preserve organic food at home.

**TABLE A3:** Items and Sub‐dimensions of the Environmental Literacy Scale for Adults (ELSA) (Atabek‐Yiğit et al. 2014).

**Environmental Consciousness Level**
Item 1: I believe that government should support the renewable energy sources (sun, wind, water, geothermal).
Item 2: Environmental education should be given from the beginning of elementary education in order to provide environmental awareness.
Item 3: I, as well as others, have responsibility for the protection of the environment.
Item 4: I'm in favor of using solar power in traffic lights and street lamps in order to keep the future generations' life.
Item 5: I'm in favor of using energy sources like solar power and natural gas since the gases given out from stoves are more harmful.
Item 6: I would use recycling boxes if there were any.
Item 7: I would use e‐bill in order to protect the environment.
Item 8: I would throw away my garbage if there were nobody there. (−)
Item 9: There is nothing wrong with pouring waste cooking oil into the sink. (−)
**Environmental Anxiety Level**
Item 10: I think we will not find a place to have picnic within a few generation.
Item 11: I think everybody should sow a tree in his or her life.
Item 12: I think seeds should be kept for the future of life.
Item 13: I would throw old newspapers; empty glass‐plastic bottles, and cans to recycling boxes.
Item 14: I think indiscriminate hunting can cause environmental problems.
Item 15: I would warn people if they caused harm to the environment.
**Environmental Awareness Level**
Item 16: When I read a newspaper I pay attention to the topics related to the environment.
Item 17: For the protection of environment caused by waste, I watch TV programs that give information about re‐use of them.
Item 18: I would like to learn about environmental issues.
Item 19: I would rather buy environmentally friendly items than economic ones.
Item 20: I prefer to use public transportation rather than private transportation to protect the environment.

**TABLE A4:** Items and sub‐dimensions of the Food Wasting Behaviors Questionnaire (FWBQ) (Misiak et al. 2023; Kendirli et al. 2025).

**Discarding unpalatable foods**
1. I throw away food I do not like.
2. I throw away dried out food.
3. I throw away wilted food.
4. I throw away food that looks unpalatable.
5. I throw away bruised food.
6. I throw away food that has been damaged.
**Buying as needed**
7. I only buy the food I need.
8. I buy small amounts of food.
9. I buy as much food as I need at any one time.
10. I avoid overshopping.
11. If possible, I buy smaller packages of food.
12. I try to buy as much food as I need at any one time.
**Planning meals**
13. I plan meals for the next day.
14. I make a plan before shopping.
15. I try to prepare meals that I can eat the next day.
16. I make shopping lists before I go grocery shopping.
17. I plan my meals.
18. I shop according to a shopping list.
**Sharing food**
19. I share the food I can't eat with people I know.
20. I give away food I can't eat.
21. I give away surplus food to people who need it.
22. I pass surplus food to people who are hungry.
23. I share food I can't eat with others.
24. I share food I can't eat with people who need it.
**Feeding animals**
25. I feed leftover food to my pets.
26. I feed leftover food to animals.
27. I feed animals when I have excess food.
28. I feed hungry animals with food I cannot eat.
29. I feed leftover food to pets.
30. I use food I have not managed to eat as fertilizer.

## References

[fsn372210-bib-0001] Ahmed, N. , C. Li , A. Khan , S. A. Qalati , S. Naz , and F. Rana . 2021. “Purchase Intention Toward Organic Food Among Young Consumers Using Theory of Planned Behavior: Role of Environmental Concerns and Environmental Awareness.” Journal of Environmental Planning and Management 64, no. 5: 796–822. 10.1080/09640568.2020.1785404.

[fsn372210-bib-0002] Ajzen, I. 1991. “The Theory of Planned Behavior.” Organizational Behavior and Human Decision Processes 50, no. 2: 179–211. 10.1016/0749-5978(91)90020-T.

[fsn372210-bib-0003] Alessandroni, L. , S. Scortichini , G. Caprioli , et al. 2024. “Assessing Chemical, Microbiological and Sensorial Shelf‐Life Markers to Study Chicken Meat Quality Within Divergent Production Systems (Organic vs. Conventional).” European Food Research and Technology 250, no. 3: 771–783. 10.1007/s00217-023-04419-2.

[fsn372210-bib-0004] Aprile, M. C. , and D. Fiorillo . 2023. “Other‐Regarding Preferences in Pro‐Environmental Behaviours: Empirical Analysis and Policy Implications of Organic and Local Food Products Purchasing in Italy.” Journal of Environmental Management 343: 118174. 10.1016/j.jenvman.2023.118174. 37247548

[fsn372210-bib-0005] Ariyani, L. , and K. R. Ririh . 2020. “Understanding Behavior of Household Food Waste Management: Food Waste Hierarchy Context.” Jurnal Ilmiah Teknologi Industri 19, no. 2: 142–154. 10.23917/jiti.v19i2.11994.

[fsn372210-bib-0006] Aschemann‐Witzel, J. , I. De Hooge , P. Amani , T. Bech‐Larsen , and M. Oostindjer . 2015. “Consumer‐Related Food Waste: Causes and Potential for Action.” Sustainability 7, no. 6: 6457–6477. 10.3390/su7066457.

[fsn372210-bib-0007] Atabek‐Yiğit, E. , N. Köklükaya , M. Yavuz , and E. Demirhan . 2014. “Development and Validation of Environmental Literacy Scale for Adults (ELSA).” Journal of Baltic Science Education 13, no. 3: 425–435. 10.33225/jbse/14.13.425.

[fsn372210-bib-0008] Attiq, S. , M. D. Habib , P. Kaur , M. J. S. Hasni , and A. Dhir . 2021. “Drivers of Food Waste Reduction Behaviour in the Household Context.” Food Quality and Preference 94: 104300. 10.1016/j.foodqual.2021.104300.

[fsn372210-bib-0009] Chauhan, K. , and A. Rao . 2024. “Clean‐Label Alternatives for Food Preservation: An Emerging Trend.” Heliyon 10, no. 16: e35815. 10.1016/j.heliyon.2024.e35815. 39247286 PMC11379619

[fsn372210-bib-0010] Cooreman‐Algoed, M. , F. Minnens , L. Boone , et al. 2021. “Consumer and Food Product Determinants of Food Wasting: A Case Study on Chicken Meat.” Sustainability 13, no. 13: 7027. 10.3390/su13137027.

[fsn372210-bib-0011] Csambalik, L. , S. A. Hollinetz , and A. Divéky‐Ertsey . 2023. “Changes of Physical and Chemical Parameters of Organic and Conventional Carrots During Storage.” Analecta Technica Szegedinensia 17, no. 4: 46–50. 10.14232/analecta.2023.4.46-50.

[fsn372210-bib-0012] Deliberador, L. R. , A. B. Santos , G. A. Queiroz , A. D. S. César , and M. O. Batalha . 2024. “The Influence of Organic Food Purchase Intention on Household Food Waste: Insights From Brazil.” Sustainability 16, no. 9: 3795. 10.3390/su16093795.

[fsn372210-bib-0013] Dusoruth, V. , and H. H. Peterson . 2020. “Food Waste Tendencies: Behavioral Response to Cosmetic Deterioration of Food.” PLoS One 15, no. 5: e0233287. 10.1371/journal.pone.0233287. 32469982 PMC7259764

[fsn372210-bib-0014] ElHaffar, G. , F. Durif , and L. Dubé . 2020. “Towards Closing the Attitude‐Intention‐Behavior Gap in Green Consumption: A Narrative Review of the Literature and an Overview of Future Research Directions.” Journal of Cleaner Production 275: 122556. 10.1016/j.jclepro.2020.122556.

[fsn372210-bib-0015] Eriksson, M. , I. Strid , and P.‐A. Hansson . 2014. “Waste of Organic and Conventional Meat and Dairy Products—A Case Study From Swedish Retail.” Resources, Conservation and Recycling 83: 44–52. 10.1016/j.resconrec.2013.11.011.

[fsn372210-bib-0016] Etim, E. , K. T. Choedron , O. Ajai , O. Duke , and H. E. Jijingi . 2025. “Systematic Review of Factors Influencing Household Food Waste Behaviour: Applying the Theory of Planned Behaviour.” Waste Management & Research, Journal of the International Solid Wastes and Public Cleansing Association, ISWA 43, no. 6: 803–827. 10.1177/0734242X241285423. PMC12106936 39385555

[fsn372210-bib-0017] Gage, E. , X. Wang , B. Xu , et al. 2024. “Reducing Food Loss and Waste Contributes to Energy, Economic and Environmental Sustainability.” Journal of Cleaner Production 451: 142068. 10.1016/j.jclepro.2024.142068.

[fsn372210-bib-0018] Gamage, A. , R. Gangahagedara , J. Gamage , et al. 2023. “Role of Organic Farming for Achieving Sustainability in Agriculture.” Farming System 1, no. 1: 100005. 10.1016/j.farsys.2023.100005.

[fsn372210-bib-0019] Gimenez, A. , G. Ares , and S. R. Jaeger . 2023. “Exploration of Individual Factors Influencing Self‐Reported Household Food Waste in Australia.” Journal of Sensory Studies 38, no. 6: e12881. 10.1111/joss.12881.

[fsn372210-bib-0020] Grasso, A. C. , M. R. Olthof , A. J. Boevé , C. Van Dooren , L. Lähteenmäki , and I. A. Brouwer . 2019. “Socio‐Demographic Predictors of Food Waste Behavior in Denmark and Spain.” Sustainability 11, no. 12: 3244. 10.3390/su11123244.

[fsn372210-bib-0021] Gray, K. 2018. “From Content Knowledge to Community Change: A Review of Representations of Environmental Health Literacy.” International Journal of Environmental Research and Public Health 15, no. 3: 466. 10.3390/ijerph15030466. 29518955 PMC5877011

[fsn372210-bib-0022] Hamzaoğlu, N. M. , and B. Öztürk Göktuna . 2022. “Food Waste Behavior of Organic Food Consumers in Turkey.” Önetim Ve Ekonomi Araştırmaları Dergisi 20, no. 4: 209–224. 10.11611/yead.1195595.

[fsn372210-bib-0023] Herzberg, R. , T. G. Schmidt , and F. Schneider . 2020. “Characteristics and Determinants of Domestic Food Waste: A Representative Diary Study Across Germany.” Sustainability 12, no. 11: 4702. 10.3390/su12114702.

[fsn372210-bib-0024] Hingston, S. T. , and T. J. Noseworthy . 2020. “On the Epidemic of Food Waste: Idealized Prototypes and the Aversion to Misshapen Fruits and Vegetables.” Food Quality and Preference 86: 103999. 10.1016/j.foodqual.2020.103999.

[fsn372210-bib-0025] Hollweg, K. S. , J. R. Taylor , R. W. Bybee , T. J. Marcinkowski , W. C. McBeth , and P. Zoido . 2011. Developing a Framework for Assessing Environmental Literacy. North American Association for Environmental Education. http://www.naaee.net.

[fsn372210-bib-0026] Hussain, M. A. , L. Li , A. Kalu , X. Wu , and N. Naumovski . 2025. “Sustainable Food Security and Nutritional Challenges.” Sustainability 17, no. 3: 874. 10.3390/su17030874.

[fsn372210-bib-0027] Icli, G. , N. K. Anil , and B. Kiliç . 2019. “Organic Food Consumption Scale (OFC): Development and Validation.” Pacific Business Review International 11, no. 9.

[fsn372210-bib-0028] Ilakovac, B. , N. Voca , L. Pezo , and M. Cerjak . 2020. “Quantification and Determination of Household Food Waste and Its Relation to Sociodemographic Characteristics in Croatia.” Waste Management 102: 231–240. 10.1016/j.wasman.2019.10.042. 31683079

[fsn372210-bib-0029] Janssens, K. , W. Lambrechts , A. Van Osch , and J. Semeijn . 2019. “How Consumer Behavior in Daily Food Provisioning Affects Food Waste at Household Level in The Netherlands.” Food 8, no. 10: 428. 10.3390/foods8100428. PMC6836184 31547123

[fsn372210-bib-0030] Joint FAO/WHO Codex Alimentarius Commission , Food and Agriculture Organization of the United Nations , and World Health Organization . 2007. “Organically Produced Foods.” In Codex Alimentarius, 3rd ed. World Health Organization: Food and Agriculture Organization of the United Nations.

[fsn372210-bib-0031] Jurgilevich, A. , T. Birge , J. Kentala‐Lehtonen , et al. 2016. “Transition Towards Circular Economy in the Food System.” Sustainability 8, no. 1: 69. 10.3390/su8010069.

[fsn372210-bib-0032] Karanth, S. , S. Feng , D. Patra , and A. K. Pradhan . 2023. “Linking Microbial Contamination to Food Spoilage and Food Waste: The Role of Smart Packaging, Spoilage Risk Assessments, and Date Labeling.” Frontiers in Microbiology 14: 1198124. 10.3389/fmicb.2023.1198124. 37426008 PMC10325786

[fsn372210-bib-0033] Kaya, V. H. , and D. Elster . 2019. “A Critical Consideration of Environmental Literacy: Concepts, Contexts, and Competencies.” Sustainability 11, no. 6: 1581. 10.3390/su11061581.

[fsn372210-bib-0034] Kendirli, G. , G. Aytekin‐Sahin , O. Mengi Celik , and M. Misiak . 2025. “Food Wasting Behaviour in Türkiye: Adaptation of the Food Wasting Behaviours Questionnaire.” British Journal of Nutrition 134, no. 3: 229–238. 10.1017/S0007114525104108. 40785586

[fsn372210-bib-0035] Khan, U. , and R. Dhar . 2006. “Licensing Effect in Consumer Choice.” Journal of Marketing Research 43, no. 2: 259–266. 10.1509/jmkr.43.2.259.

[fsn372210-bib-0036] Koivupuro, H. , H. Hartikainen , K. Silvennoinen , et al. 2012. “Influence of Socio‐Demographical, Behavioural and Attitudinal Factors on the Amount of Avoidable Food Waste Generated in Finnish Households.” International Journal of Consumer Studies 36, no. 2: 183–191. 10.1111/j.1470-6431.2011.01080.x.

[fsn372210-bib-0037] Kritikou, T. , D. Panagiotakos , K. Abeliotis , and K. Lasaridi . 2021. “Investigating the Determinants of Greek Households Food Waste Prevention Behaviour.” Sustainability 13, no. 20: 11451. 10.3390/su132011451.

[fsn372210-bib-0038] Li, X. , Y. Jiang , and P. Qing . 2023. “Estimates of Household Food Waste by Categories and Their Determinants: Evidence From China.” Food 12, no. 4: 776. 10.3390/foods12040776. PMC9956864 36832853

[fsn372210-bib-0039] Lima, R. , A. Yu , Q. Liu , and J. Liu . 2024. “Examining the Determinants of Food Waste Behavior in China at the Consumer Level.” Food Security 16, no. 4: 867–881. 10.1007/s12571-024-01466-9.

[fsn372210-bib-0040] Lovren, V. O. , and M. M. Jablanovic . 2023. “Bridging the Gap: The Affective Dimension of Learning Outcomes in Environmental Primary and Secondary Education.” Sustainability 15, no. 8: 6370. 10.3390/su15086370.

[fsn372210-bib-0041] Mazar, N. , and C.‐B. Zhong . 2010. “Do Green Products Make Us Better People?” Psychological Science 21, no. 4: 494–498. 10.1177/0956797610363538. 20424089

[fsn372210-bib-0042] Melbye, E. L. , Y. Onozaka , and H. Hansen . 2017. “Throwing It All Away: Exploring Affluent Consumers’ Attitudes Toward Wasting Edible Food.” Journal of Food Products Marketing 23, no. 4: 416–429. 10.1080/10454446.2015.1048017.

[fsn372210-bib-0043] Melovic, B. , D. Cirovic , B. Dudic , T. B. Vulic , and M. Gregus . 2020. “The Analysis of Marketing Factors Influencing Consumers’ Preferences and Acceptance of Organic Food Products—Recommendations for the Optimization of the Offer in a Developing Market.” Food 9, no. 3: 259. 10.3390/foods9030259. PMC7142824 32121318

[fsn372210-bib-0044] Mengi Çelik, Ö. , S. D. Akçakaya , and E. M. Ekici . 2025. “Relationship Between Sustainable Food Literacy, Organic Food Consumption and Climate Change Awareness and Worry in Türkiye.” BMC Public Health 25, no. 1: 2491. 10.1186/s12889-025-22482-0. 40681983 PMC12273353

[fsn372210-bib-0045] Mengi Çelik, Ö. , E. M. Ekici , S. Yılmaz , and Z. E. Metin . 2025. “Evaluation of the Relationship Between Nutrition Literacy, Mediterranean Diet Compliance, Ecological Footprint and Sustainable Environmental Attitudes in Adolescents.” BMC Public Health 25, no. 1: 130. 10.1186/s12889-024-20910-1. 39800704 PMC11727382

[fsn372210-bib-0046] Misiak, M. , M. Sobol , L. Sakowski , M. Kowal , A. Jurczyk , and L. Wojtycka . 2023. “Five Ways to Waste Food: Food Wasting Behaviours Questionnaire.” British Food Journal 125, no. 9: 3437–3455. 10.1108/BFJ-07-2022-0641.

[fsn372210-bib-0047] Nadricka, K. , K. Millet , and A. Aydinli . 2024. “Are Consumers More or Less Averse to Wasting Organic Food?” Journal of Environmental Psychology 93: 102222. 10.1016/j.jenvp.2023.102222.

[fsn372210-bib-0048] Oktaviani, R. D. , P. Naruetharadhol , S. Padthar , and C. Ketkaew . 2024. “Green Consumer Profiling and Online Shopping of Imperfect Foods: Extending UTAUT With Web‐Based Label Quality for Misshapen Organic Produce.” Food 13, no. 9: 1401. 10.3390/foods13091401. PMC11083663 38731772

[fsn372210-bib-0049] Parashar, S. , S. Singh , and G. Sood . 2023. “Examining the Role of Health Consciousness, Environmental Awareness and Intention on Purchase of Organic Food: A Moderated Model of Attitude.” Journal of Cleaner Production 386: 135553. 10.1016/j.jclepro.2022.135553.

[fsn372210-bib-0050] Parsa, A. , M. Van De Wiel , U. Schmutz , J. Fried , D. Black , and I. Roderick . 2023. “Challenging the Food Waste Hierarchy.” Journal of Environmental Management 344: 118554. 10.1016/j.jenvman.2023.118554. 37406496

[fsn372210-bib-0051] Paull, J. 2010. “From France to the World: The International Federation of Organic Agriculture Movements (IFOAM).” Journal of Social Research and Policy 1, no. 2.

[fsn372210-bib-0052] Pilone, V. , N. Di Santo , and R. Sisto . 2023. “Factors Affecting Food Waste: A Bibliometric Review on the Household Behaviors.” PLoS One 18, no. 7: e0289323. 10.1371/journal.pone.0289323. 37506105 PMC10381066

[fsn372210-bib-0053] Price, G. , and G. Clark . 2024. “The Hidden Climate Cost: Food Loss, Waste, and Greenhouse Gas Emissions.” Open Access Gov 44, no. 1: 436–437. 10.56367/OAG-044-11589.

[fsn372210-bib-0054] Principato, L. , L. Secondi , and C. A. Pratesi . 2015. “Reducing Food Waste: An Investigation on the Behaviour of Italian Youths.” British Food Journal 117, no. 2: 731–748. 10.1108/BFJ-10-2013-0314.

[fsn372210-bib-0055] Przezbórska‐Skobiej, L. , and P. L. Wiza . 2021. “Food Waste in Households in Poland—Attitudes of Young and Older Consumers Towards the Phenomenon of Food Waste as Demonstrated by Students and Lecturers of PULS.” Sustainability 13, no. 7: 3601. 10.3390/su13073601.

[fsn372210-bib-0056] Puteri, B. , B. Buttlar , and B. Jahnke . 2022. “Take It or Leave It? Investigating the Ambivalence and Willingness to Pay for Suboptimal Fruits and Vegetables Among Organic Consumers in Germany.” Frontiers in Sustainable Food Systems 6. 10.3389/fsufs.2022.934954.

[fsn372210-bib-0057] Rana, J. , and J. Paul . 2020. “Health Motive and the Purchase of Organic Food: A Meta‐Analytic Review.” International Journal of Consumer Studies 44, no. 2: 162–171. 10.1111/ijcs.12556.

[fsn372210-bib-0058] Romani, S. , S. Grappi , R. P. Bagozzi , and A. M. Barone . 2018. “Domestic Food Practices: A Study of Food Management Behaviors and the Role of Food Preparation Planning in Reducing Waste.” Appetite 121: 215–227. 10.1016/j.appet.2017.11.093. 29155173

[fsn372210-bib-0059] Roth, C. E. 1992. Environmental Literacy: Its Roots, Evolution and Directions in the 1990s’, ERIC/CSMEE Publications. Ohio State University. https://eric.ed.gov/?id=ED348235.

[fsn372210-bib-0060] Sachs, J. D. , G. Lafortune , G. Fuller , and G. Iablonovski . 2025. Sustainable Development Report 2025 Financing Sustainable Development to 2030 and Mid‐Century. Dublin University Press. 10.25546/111909.

[fsn372210-bib-0061] Shalini, J. R. , G. S. Sreedaya , and R. Mathew . 2025. “Transforming Global Food Systems: Pathways to Sustainability, Resilience and Equity.” Archives of Current Research International 25, no. 3: 145–154. 10.9734/acri/2025/v25i31104.

[fsn372210-bib-0062] Sousa, R. D. , L. Bragança , M. V. Da Silva , and R. S. Oliveira . 2024. “Challenges and Solutions for Sustainable Food Systems: The Potential of Home Hydroponics.” Sustainability 16, no. 2: 817. 10.3390/su16020817.

[fsn372210-bib-0063] Srijuntrapun, P. , P. Ket‐um , and W. Attavanich . 2025. “Socio‐Demographic Drivers of Household Food Waste Management Practices in Thailand.” PLoS One 20, no. 4: e0321054. 10.1371/journal.pone.0321054. 40168395 PMC11960981

[fsn372210-bib-0064] Stern, P. C. 2000. “New Environmental Theories: Toward a Coherent Theory of Environmentally Significant Behavior.” Journal of Social Issues 56, no. 3: 407–424. 10.1111/0022-4537.00175.

[fsn372210-bib-0065] Stoica, D. , A. E. Micu , and M. Stoica . 2025. “Factors That Influence the Household Food Wastes.” Journal of Interdisciplinary Cross‐Border Studies 5, no. 3. 10.35219/across.2022.5.3.04.

[fsn372210-bib-0066] Szymkowiak, A. , B. Borusiak , B. Pierański , P. Kotyza , and L. Smutka . 2022. “Household Food Waste: The Meaning of Product's Attributes and Food‐Related Lifestyle.” Frontiers in Environmental Science 10: 918485. 10.3389/fenvs.2022.918485.

[fsn372210-bib-0067] Teixeira, S. F. , B. Barbosa , H. Cunha , and Z. Oliveira . 2021. “Exploring the Antecedents of Organic Food Purchase Intention: An Extension of the Theory of Planned Behavior.” Sustainability 14, no. 1: 242. 10.3390/su14010242.

[fsn372210-bib-0068] Tonini, P. , P. M. Odina , and X. G. Durany . 2023. “Predicting Food Waste in Households With Children: Socio‐Economic and Food‐Related Behavior Factors.” Frontiers in Nutrition 10: 1249310. 10.3389/fnut.2023.1249310. 37876618 PMC10591224

[fsn372210-bib-0069] United Nations Department of Economic and Social Affairs . 2023. “The Sustainable Development Goals Report 2023: Special Edition.” In The Sustainable Development Goals Report. United Nations. 10.18356/9789210024914.

[fsn372210-bib-0070] United Nations Environment Programme . 2024. “Food Waste Index Report 2024. In Think Eat Save: Tracking Progress to Halve Global Food Waste.” United Nations Environment Programme. https://wedocs.unep.org/handle/20.500.11822/45230.

[fsn372210-bib-0071] Varjani, S. , S. Vyas , J. Su , et al. 2024. “Nexus of Food Waste and Climate Change Framework: Unravelling the Links Between Impacts, Projections, and Emissions.” Environmental Pollution 344: 123387. 10.1016/j.envpol.2024.123387. 38242308

[fsn372210-bib-0072] Varzakas, T. , and S. Smaoui . 2024. “Global Food Security and Sustainability Issues: The Road to 2030 From Nutrition and Sustainable Healthy Diets to Food Systems Change.” Food 13, no. 2: 306. 10.3390/foods13020306. PMC10815419 38254606

[fsn372210-bib-0073] von Braun, J. , K. Afsana , L. O. Fresco , and M. H. A. Hassan . 2023. “Food Systems: Seven Priorities to End Hunger and Protect the Planet.” In Science and Innovations for Food Systems Transformation, edited by J. Von Braun , K. Afsana , L. O. Fresco , and M. H. A. Hassan , 3–9. Springer International Publishing. 10.1007/978-3-031-15703-5_1.

[fsn372210-bib-0074] WHO . 2025. “Body Mass Index (BMI).” https://www.who.int/data/gho/data/themes/topics/topic‐details/GHO/body‐mass‐index.

[fsn372210-bib-0075] Xu, M. , Q. Xu , S. Wei , X. Gu , and F. Liu . 2024. “Modulating Variables Impacting the Intersection of Health and Environmental Concerns in Organic Food Purchasing Decisions.” British Food Journal 126, no. 10: 3663–3683. 10.1108/BFJ-09-2023-0849.

[fsn372210-bib-0076] Yildirim, M. S. , A. Elkoca , G. Gökçay , D. A. Yilmaz , and M. Yıldız . 2025. “The Relationship Between Environmental Literacy, Ecological Footprint Awareness, and Environmental Behavior in Adults.” BMC Public Health 25, no. 1: 551. 10.1186/s12889-025-21340-3. 39930452 PMC11812180

